# The Pharmacological Effects of Morroniside and Loganin Isolated from Liuweidihuang Wan, on MC3T3-E1 Cells

**DOI:** 10.3390/molecules15107403

**Published:** 2010-10-21

**Authors:** Manyu Li, Wei Wang, Ping Wang, Kun Yang, Hui Sun, Xijun Wang

**Affiliations:** National TCM Key Lab of Serum Pharmacochemistry, Heilongjiang University of Chinese Medicine, Heping Road 24, Harbin 150040, China

**Keywords:** Liuweidihuang Wan, morroniside, loganin, osteoporosis, MC3T3-E1

## Abstract

Liuweidihuang Wan (LW), initially a well-known formula for curing “wu chi wu ruan”, is commonly used nowadays for clinical treatment of Postmenopausal Osteoporosis (PO), but the identity of the effective substance(s) remains unclear. The present study was designed to evaluate the effects of morroniside and loganin isolated from LW on the proliferation, differentiation and apoptosis of MC3T3-E1 cells, as well as the possible mechanism of action. Morroniside and loganin had no effects on the proliferation of MC3T3-E1 cells, but both susbtances could improve the activity of alkaline phosphatase (ALP), and increase the contents of collagen type I and osteocalcin. Simultaneously, the mRNA expression of caspase-3, capase-9, RANKL was down-regulated and that of bcl-2 was up-regulated, which partially explains the anti-osteoporosis mechanism in MC3T3-E1 cells. In conclusion, morroniside and loganin may directly promote the differentiation and inhibit the apoptosis of MC3T3-E1 cells, and accordingly indirectly reduce bone resorption, which makes them promising natural drugs leads for treating PO in the near future.

## 1. Introduction

Traditional Chinese medicine (TCM) has been used clinically in China and Asia for more than 8,000 years. TCM has been modified to some extent in other countries, such as in Korea and Japan, and has recently attracted serious interest in Europe and North America. Osteoporosis is a multi-factorial bone disease characterized by reduction of bone mass, damage to the bone tissue microstructure, aggravation of osteopsathyrosis and increased risk of fractures [1]. It is one of the most common diseases in elderly patients and represents a major public health problem, as more than 50% of postmenopausal women and 20% of men older than 50 years suffer from osteoporosis [2]. According to statistics, there are over 200 million victims worldwide, with some 75 million in Europe, Japan and the US [2,3]. Its main clinical feature are fractures, which usually occur in the forearm, spine and hip, and each year more than 150 million people suffer from the fractures of hip, spine and wrist caused by osteoporosis. Moreover, the incidence of fractures in other body regions may be related to osteoporosis too. Osteoporotic fractures can lead to high mortality and disability, reduce the flexibility of the body and quality of life [4]. Up to 50% of women who sustain hip fractures afterwards need auxiliary equipment to carry out their daily life activities, about 20% will die within one year, and there are a considerable number of people requiring long-term care [5,6]. In addition, people with one or more vertebral fractures easily suffer from osteoporosis and the general aging of the population is bound to increase the incidence of osteoporotic fractures. In view of its seriousness, the prevention and treatment of osteoporosis will be one of the main tasks for all the national health agencies [7]. Many risk factors are related with osteoporosis, including diabetes, hormonal factors, glucocorticoids, smoking, sedentary lifestyle, low calcium and vitamin D intake as well as a personal or family history of fractures. Osteoporosis occurs as a result of multiple mechanisms and the combined actions of loss of bone mass and decrease of bone strength [8]. In women, osteoporotic fractures mainly occur as a consequence of estrogen deficiency after menopause and an imbalance between bone resorption caused by osteoclasts and bone formation caused by osteoblasts, leading to a net bone loss in each remodeling cycle [9]. 

Pharmacotherapy is a major therapeutic option for osteoporosis, with conventional medicines including calcium, vitamin D, estrogen supplements, biphosphonates, calcitonin and TCMs. Hormone replacement therapy has some adverse reactions which can cause uterine cancer and breast cancer, increased incidence of bile duct stones and elevated blood lipids. The adverse reactions of bisphosphonates are mainly gastrointestinal reactions, such as nausea, vomiting, abdominal pain and diarrhea. Low conversion-type osteoporosis patients in long-term bisphosphonate treatment regimes can experience reduction of new bone formation, a relative increase of the old bone mass and bone deterioration. Taking calcium and vitamin D has little effect on osteoporosis, and the prevention for various types of fractures does not have systemic effects, but may increase the risk of kidney stones. 

MC3T3-E1 cell is a clonal osteoblastic cell line isolated from C57BL/6 mouse calvaria. Osteoblasts are the most significant cells in bone tissue, and are the key points for bone formation and maintenance of bone mass. Differentiation of osteoblasts mainly includes three steps – division growth, ground substance maturation and mineralization. MC3T3-E1 cells are adherent cells and selected in most osteoblast studies, as the cells are typical, easy to culture, have a clearly understood mechanism and are stable enough for reproducibility. All these features meet our experimental requirements, therefore, MC3T3-E1 cells were selected in the present study. The Korean *Fructus Corni*-containing herbal medicine “Yukmi-jihang-tang” (YJ) has anti-bone resorption characteristics, and as used to treat reduction of bone mass caused by diabetes [10]. In China, LW, which also contains *Fructus Corni*, has a long history and wide application in the clinic, initially for the treatment of “wu chi wu ruan” of children, and nowadays for treating osteoporosis in postmenopausal women or elderly men. The aqueous extract of *Fructus Corni* and total glycosides could also increase the total bone density in mice and enhance the mechanical properties, but so far, the identity of the pharmacodynamic material(s) in isolated “*Fructus Corni*” herbs and the compound recipes used for the treatment of osteoporosis is unknown. Morroniside and loganin can be detected not only in the water and alcohol extracts of *Fructus Corni,* but also in rat serum after administration with LW, and thus they could be used as biomarkers to evaluate the therapeutic effect of the formula [11]. The present study was undertaken to find out if morroniside and loganin isolated form *Fructus Corni* had any effects on proliferation, differentiation and apoptosis of MC3T3-E1 and RANKL system, and to investigate the possible mechanism on a molecular level.

## 2. Results 

### 2.1. The effects of morroniside and loganin on the proliferation of MC3T3-E1 Cells

**Figure 1:**
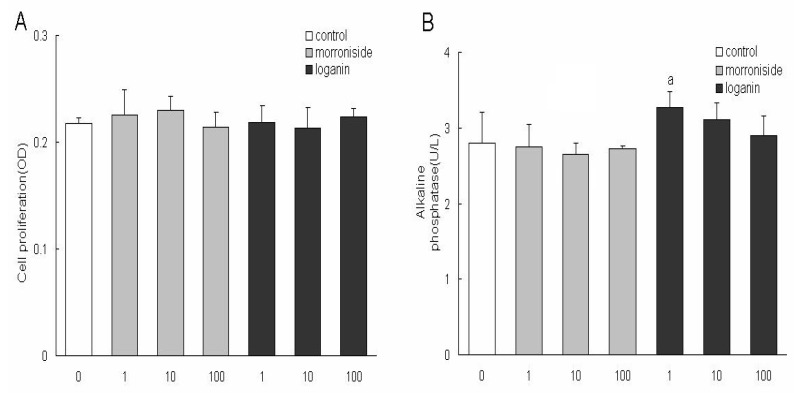

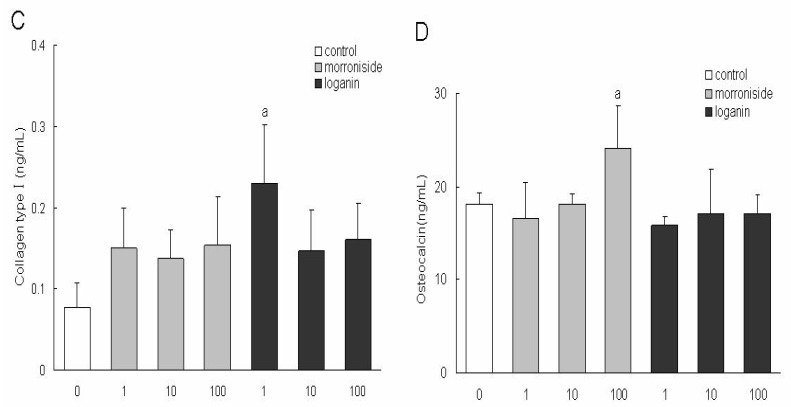
The effects of morronside and loganin on the proliferation of MC3T3-E1 cells. Cells were cultured with medium containing different concentrations (1, 10, 100 μg/mL) of the two constituents for 96 h and the viability of cells was measured by MTT method; **B:** The activity of ALP in MC3T3-E1 cells; **C:** The contents of osteocalcin in MC3T3-E1 cells; **D:** The contents of collagen type I in MC3T3-E1 cells.

To investigate the effects of morroniside and loganin on the proliferation of MC3T3-E1 cells, cells were firstly treated with different concentrations of the two constituents at 1, 10, 100 μg/mL respectively for 96 h and the viability of cells was measured by MTT method. Neither of them showed significant effects on cell growth compared with that in control one, indicating that the two constituents had no toxic effect at the three concentrations on the cells, therefore, the final concentrations were determined at 1, 10, 100 μg/mL in the following experiments (Figure 1A).

### 2.2. Effects of morroniside and loganin on the activity of ALP, contents of osteocalcin and collagen type I in MC3T3-E1 Cells

To investigate the effects of morroniside and loganin on the differentiation of MC3T3-E1 cells, cells were first treated with different concentrations of the two constituents at 1, 10, 100 μg/mL, respectively, for 48 h and the activity of ALP, contents of osteocalcin and collagen type I were detected. Only after 48 h of incubation with loganin at 1 μg/mL, the activity of ALP, contents of collagen type I were increased significantly, but they were not increased after pretreatment with morroniside at any concentration (Figure 1B, Figure 1C). The effects of morroniside and loganin on the terminal differentiation of MC3T3-E1 cells were also assessed by determining the contents of osteocalcin. Morroniside at 100 μg/mL increased the content of osteocalcin in a seemingly dose-dependent manner (Figure 1D).

### 2.3. Effects of morroniside and loganin on mRNA expression of caspase-3, caspase-9, bcl-2 and RANKL in MC3T3-E1 cells

**Figure 2:**
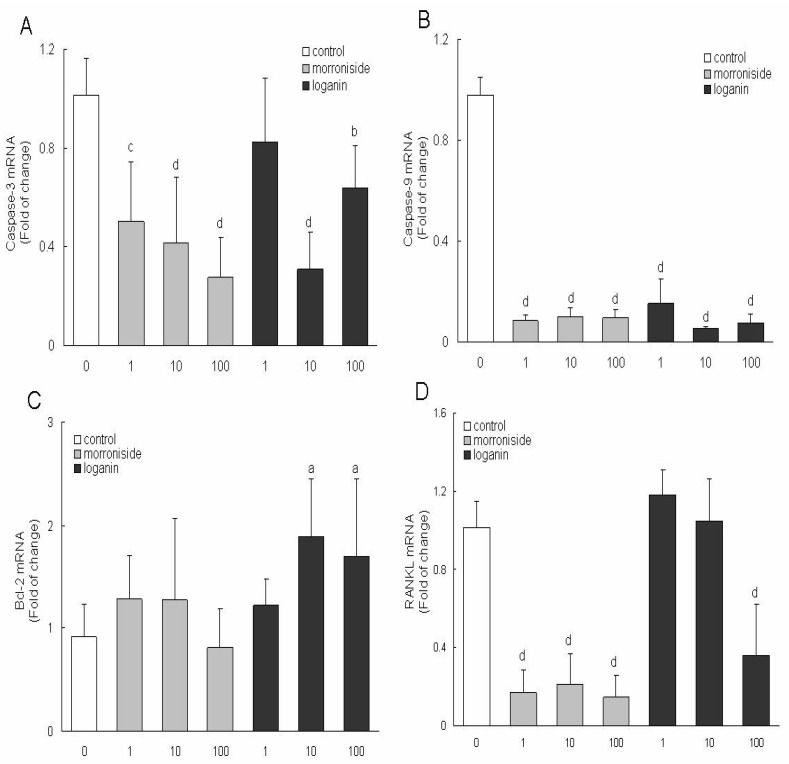
The amounts of caspase-3 mRNA in MC3T3-E1 cells; **B:** The amounts of caspase-9 mRNA in MC3T3-E1 cells; **C:** The amounts of bcl-2 mRNA in MC3T3-E1 cells; **D:** The amounts of RANKL mRNA in MC3T3-E1 cells.

MC3T3-E1 cells were treated with various concentrations of morroniside and loganin for 48 h, and then the amounts of caspase-3, caspase-9, bcl-2 and RANKL mRNA were assessed by RT-PCR (Figure 2A-D). The amounts of PCR products of caspase-3 mRNA were significantly decreased after cells were treated with different concentrations of morroniside (1, 10, 100 μg/mL) and loganin (10, 100 μg/mL) and those of caspase-9 mRNA were significantly decreased after cells were treated with different concentrations of morroniside (1, 10, 100 μg/mL) and loganin (1, 10, 100 μg/mL). On the other hand, the expressions of bcl-2 mRNA were obviously up-regulated only after cells treated with loganin (10, 100 μg/mL) and that RANKL mRNA were down-regulated by morroniside (1, 10, 100 μg/mL) and loganin at 100 μg/mL. The results suggested that morroniside and loganin could promote differentiation and inhibit apoptosis of MC3T3-E1 cells, and could also down-regulated the expression of RANKL mRNA, so that the role of osteolysis was inhibited and the function of bone was improved, which may partly explain the mechanism of the two constituents on osteoporosis.

## 3. Discussion

LW, a well-known formula used initially for curing “wu chi wu ruan”, was first recorded in “*Xiao er Yao Zheng Zhi Jue*” as consisting of *Radix Rehmanniae Preparata, Fructus Corni, Rhizoma Dioscoreae, Poria, Rhizoma Alismatis* and *Cortex Moutan* in a 8:4:4:3:3:3 proportion. Eleven chemical constituents were detected in the blood of rats after administration with the extracts of LW, and could be the effective substances of LW, according to the TCM theory of serum pharmaco-chemistry [12], according to which there are many different constituents in TCM, but not all of them are active components. If a component can play a part in the body, it should first be absorbed into the blood. In other words, the constituents that are absorbed into blood might be the active compounds and the constituents that were excreted directly cannot be the active compounds in most conditions. Morroniside and loganin, mainly contained in *Macrocarpium officinale*, had protective effects in the rat model of shen deficiency induced by hydrocortisone [13]. It is also reported that LW could improve the bone density of lumbar vertebrae, femur neck and radius in PO patients, and increase the level of ALP and osteocalcin, reducing the activity of TRAP [14]. The contents of calcium and phosphorus in the bone were increased and the load and deflection were markedly improved after ovariectomized rats administrated with LW [15]. The aqueous extract of *Fructus Corni* could increase the depth of cortical bone and the area of bone trabeculae in SAM-P/6 mice [16]. 

Morroniside and loganin are the two main constituents of the eleven plasma effective constituents, mainly contained in *Macrocarpium officinale*, so they were isolated from *Macrocarpium officinale* in the present study. Morroniside (1, 10, 100 μg/mL) or loganin (1, 10, 100 μg/mL) had no effect on the growth of MC3T3 cells, therefore, the two constituents at determined concentrations were used to treat the MC3T3 cells in the following experiments. The present study is the first to demonstrate that morroniside and loganin induced maturation and differentiation of MC3T3 cells, without effects on cell growth. The results indicated that loganin could increase the production of ALP and collagen type and morroniside could increase the production of osteocalcin. As ALP is an early phenotypic marker for mature osteoblasts, the results suggested that both of morroniside and loganin could stimulate the differentiation of osteoblast in an early stage. In contrast, osteocalcin is a phenotypic marker for differentiation of osteoblast in a later stage, one that coincides with mineralization, and it is one of the major noncollagenous proteins specific to mineralized connective tissues of vertebrates [17,18]. In summary, these results indicated that morroniside and loganin stimulated maturation and differentiation of osteoblast cells from early to terminal stages in the whole differentiation process.

Apoptosis is a process with multiple steps including upstream induction phases and downstream execution stages [19,20,21,22]. The rate of bone formation and resorption is largely determined on the numbers of bone-forming (osteoblast) and bone-resorbing (osteoclast) cells present in the basic multicellular units responsible for the regeneration of the adult skeleton [23]. The number of bone cells is regulated by changes not only in the production of mature cells but also in their survival. It is demonstrated that apoptosis (programmed cell death) represents the most common fate of osteoblasts during physiologic bone remodeling [24,25]. Apoptosis is regulated by specific intracellular signaling pathways that ultimately induce cell self-destruction. The process of apoptosis is a highly ordered chain of events involving the sequential activation of different members of a family of aspartate-specific cystein proteases called caspases. These proteases are important effectors of apoptosis and are responsible for the proteolytic cleavage of selected cellular proteins, including proteins responsible for DNA repair and proteins involved in cell cycle regulation [26]. Members of the Bcl-2 protein family are proteins with the ability to modify the apoptotic pathway by affecting cytochrome release from the mitochondrion. Some members of this protein family (for instance, Bax) act as promoters of apoptosis, and others (such as Bcl-2) as inhibitors of apoptosis [27]. Caspases 9 are initiator caspases that in turn activate downstream executioner caspases, such as 3, 6 and 7. Osteoblasts and osteocytes undergoing apoptosis in response to GCs display typical features, such as activation of caspase 3 and DNA laddering [28,29]. Executioner caspases, such as caspase 3, are blocked by a family of inhibitors of apoptosis proteins, many of which are ubiquitin ligases and target the caspases for proteasomal degradation [30,31]. Receptor activator of nuclear factor kappa-B ligand (RANKL) and its receptor RANK are the key regulators of bone remodeling [32].

RANKL is a member of the TNF superfamily with high similarity to other members of that group. It is the main stimulatory factor for formation of mature osteoclasts and is essential for their survival. Therefore, the molecule is the principal final mediator of osteoclastic bone resorption. RANKL is produced by osteoblastic lineage cells and activated locally in the bone. Osteoporosis is characterized by low bone mass and microarchitectural deterioration of the skeleton, leading to an increased risk of fracture after minimal trauma. Therapeutic agents that increase the number of osteoblasts could improve bone mass and decrease the risk of fractures. Controlling cell proliferation, differentiation, and apoptosis is also important for the production of preosteoblasts and osteoblasts. 

Cell culture could mimic the growth process of osteoblasts, which is similar with that observed *in vivo* [33]. The biomarkers of osteoblasts, such as collage type I, osteocalcin, would be produced in the cell culture process. It is reported that estrogen could inhibit the proliferation of osteosarcoma cell line UMR 106 and improve the activity of ALP [34]. Knott observed that the proliferation rate of osteoblasts was lowered in the rats without double-side ovary after estrin treatment for a week [35]. These results suggested that estrogen could convert the cell proliferation to cell differentiation and improve the function of differentiation in osteoblasts. The level of estrogen will reduce rapidly in post-menopause women, and the function of inhibited proliferation is relieved, so that the number of osteoblasts is increased. However, the normal bone tissue could not be formed without the differentiation effect of estrogen and generous osteoblasts in a low differentiated degree will be apoptosis in the final. Osteoclasts have a close relationship with osteoblasts, especially in the early stage of osteoblasts [36]. The formation of generous osteoclasts will result in osteoporosis, as the disproportion between osteoclasts and osteoblasts. Osteoclasts were usually contained in cancellous bone, and the bone resorption was increased significantly after OVX [37].

## 4. Experimental

### 4.1. Morroniside and loganin preparation

**Figure 3:**
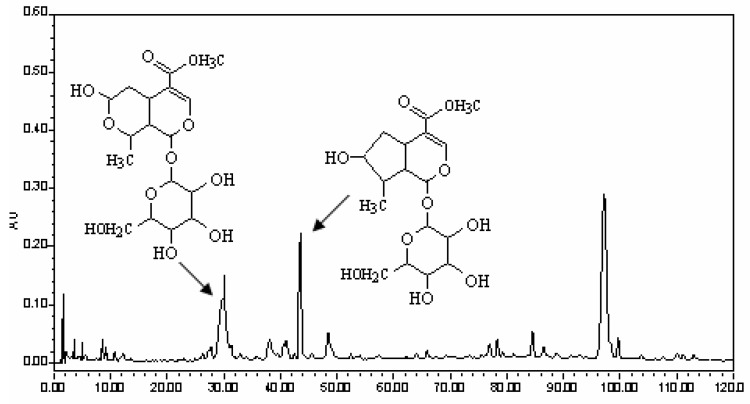
The HPLC charts of LW and the marked peaks of morronside and loganin.

The six crude drugs, *Radix Rehmanniae Preparata, Fructus Corni, Rhizoma Dioscoreae, Poria, Rhizoma Alismatis* and *Cortex Moutan*, were purchased from the Harbin Tongrentang Drugstore and were authenticated by Professor Xijun Wang of the Department of Pharmacognosy, Heilongjiang University of Chinese Medicine. The six drugs were cut into pieces, then all the pieces were powdered and passed through 100 mesh, and were made into pills according the process recorded in the China Pharmacopeia [38]. The pills were extracted twice with a 10-fold volume of methanol under ultrasound for 15 min. Finally the filtrates were combined, and solvents were removed. The residue was dissolved in 10 times the volume of methanol and the solution was used for HPLC analysis. For reversed-phase HPLC analysis, a Water Symmetry Shield RP18 column (150 mm × 3.9 mm i.d., 5 µm, Waters Corporation, Milford, MA, USA) was used. The column was eluted with a linear gradient of 1-12% A over 0.1-60.0 min, 12-30% A over 60.0-130.0 min, at an eluent flow rate of 1000 µL/min, the UV detector at 242 nm, where mobile phase A consisted of acetonitrile and mobile phase B consisted of 0.15% phosphoric acid in water. The HPLC trace of LW is shown in Figure 3 where the peaks of the two constituents were marked.

Morroniside and loganin were the main active constitutents of LW and they are mainly contained in the crude drug of *Macrocarpium officinale* according to our former studies, therefore *Macrocarpium officinale* was used for the isolation of the two compounds in the present study. Cornus pieces were extracted with 90% ethanol, and solvents were removed, then the concentrate was dissolved in water; the supernatant was subjected to macroporous resin, and 10% ethanol eluate was collected as fraction A which was separated using silica gel column chromatography (dichloromethane-methanol = 1:99, v/v; 7:93, v/v), then fractions B (5:95 v/v) and C (7:93 v/v) were obtained; fractions B and C were purified by preparative liquid chromatography (methanol-water = 40:60, v/v) in order to obtain morroniside and loganin, whose purity was ≥ 98%. 

### 4.2. Cell culture

MC3T3-E1 cells were dispensed in 96-well plates at a density of 1 × 10^6^ cells per 1 mL in basal Eagle’s medium supplemented with 10% heat-inactivated fetal bovine serum, 2 mM glutamine, gentamicin (100 mg/mL), antibiotic-antimycotic solution (10 mL/L) and 10 mM KCl, which promotes the survival of MC3T3-E1 cells at 37 ºC in a humidified 5% CO_2_ atmosphere. The medium contained morroniside or loganin was changed every 2 days and added to plates to treat the proliferation of MC3T3-E1 cells; 4 days later, the viable cells were examined by the modified MTT assay, 20 μL MTT solution (5 mg/mL) was added to each well; after incubation with MTT for 4 h, the precipitates were dissolved in 150 μL of DMSO; the cells were oscillated for 10 min, and their absorption at 490 nm was determined by the enzyme-linked immunosorbent assay instrument.

### 4.3. MTT test assay

MC3T3-E1 cells were dispensed in 96-well plates at a density of 1 × 104 cells per well at 37 ºC in a humidified 5% CO_2_ atmosphere. Then cells were treated with different concentrations of morroniside and loganin medium respectively, which was changed every 2 days; 4 days later, the viable cells were examined by the modified MTT assay, 20 μL MTT solution (5 mg/mL) was added to each well; after incubation with MTT for 4 h, the precipitates were dissolved in 150 μL of DMSO; the cells were oscillated for 10 min, and their absorption at 490 nm was determined by the enzyme-linked immunosorbent assay instrument.

### 4.4. Enzyme-linked immunosorbent assay and recording

The 3T3-E1 cells in logarithmic phase were digested with 0.25% trypsogen; cell suspension was prepared and inoculated to 24-well plates (1 × 10^4^/well). 24 h later, the culture medium containing different concentration drugs were added to each well; cultured for 2 days, the supernatant and cells was used for ELISA and PCR experiments, respectively. The determination of ALP, osteocalcin (OCN), collagen type I (I collagen) was applying ELISA (R & D Systems).

### 4.5. Real-time reverse transcriptase polymerase chain reaction (RT-PCR) analysis

Total RNA was isolated from MC3T3-E1 cells that pretreated with different concentrations of morroniside and loganin. Reverse transcription was performed with AMV reverse transcriptase (TaKaRa) and oligodT15 primers (TaKaRa). RT-PCR was carried out using SYBR Green I dye. Relative expression of the caspase-3, caspase-9, bcl-2 and RANKL genes was measured by RT-PCR using β-actin as internal control. Primer sequences were: β-actin (forward, 5′-cagccttccttcttgggt at-3′; reverse, 5′-tggcatagaggtctttacgg-3′; 100 bp expected); caspase-3 (forward, 5′-actggaaagccgaaactc -3′; reverse, 5′-gtcccactgtctgtctcaat-3′; 80 bp expected); caspase-9 (forward, 5′-ccgtggacattggttctg-3′; reverse, 5′-cac attgttgatgatgaggc-3′; 125 bp expected); bcl-2 (forward, 5′-cgggagaacagggtatga-3′; reverse, 5′-aggctggaaggagaagat-3′; 148 bp expected); RANKL (forward, 5′-atcgggaagcgtacctaca-3′; reverse, 5′-tccctcctttcatcaggttat-3′; 98 bp expected). RT-PCR was carried out with the following thermal cycling conditions: denaturation at 95 ºC for 5 min, followed by 40 cycles of 30 s at 94 ºC, 30 s at 57 ºC, and 30 s at 72 ºC (β-actin and caspase-9); denaturation at 95 ºC for 5 min, followed by 40 cycles of 30 s at 94 ºC, 30 s at 52 ºC, and 30 s at 72 ºC (RANKL and caspase-3); denaturation at 95 ºC for 5 min, followed by 40 PCR cycles of 30 s at 94 ºC, 30 s at 55 ºC, and 30 s at 72 ºC (bcl-2). Relative gene expression was presented as 2^(-△Ct)^, where △Ct=Ct_target gene_ -Ct _β-actin_. Fold change was calculated as 2^-△△Ct^, where △△Ct=△Ct_treatment_-Ct_vehicle_.

### 4.6. Statistical analysis

Each data point in the figures represents the Mean±SD of three separate experiments. Statistically significant differences between treatments and controls were determined by one-way ANOVA and then Least Significance Difference (LSD) comparison procedure using SPSS 16.0. Statistical significance was set at p < 0.05.

## 5. Conclusions

**Figure 4:**
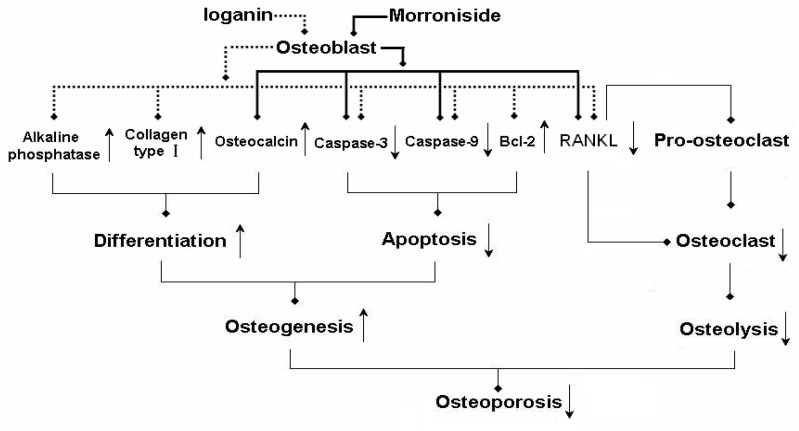
The molecular mechanism of morroniside and loganin on treating osteoporosis.

The results suggest that the mechanism of action of morroniside and loganin on osteoporosis was as follows (Figure 4): on the one hand, both of them could not only promote differentiation of osteoblasts by increasing secretion of alkaline phosphatase, osteocalcin and collagen type I, but also could inhibit apoptosis of osteoblasts by down-regulating the expressions of caspase-3 and caspase-9, up-regulating the expressions of bcl-2 to raise survival rate of osteoblasts. By promoting differentiation and inhibiting apoptosis, morroniside and loganin could enhanced osteogenesis of osteoblasts; on the other hand, morroniside and loganin could down-regulate expression of osteoblast RANKL, drawdown of which could not only decrease the number of osteoclasts from pro-osteoclasts, but also have an effect on the maturation of osteoclasts as well as suppress the bone resorption of osteoclasts. In a word, morroniside and loganin could directly enhance the function of osteoblasts and elevate their survival rate, indirectly inhibit the function of osteoclastd and reduce the number of osteoclastd via RANKL from osteoblastd. Ultimately, the unbalanced state between bone resorption and bone formation is transformed to a balanced condition in order to treat osteoporosis. Thus, alone or in combination, the morroniside and loganin agents are expected to become a new type of drug treatment for osteoporosis.

## Figures and Tables

Cells were cultured with medium containing different concentrations (1, 10, 100 μg/mL) of the two constituents for 48 h and the activity of ALP, contents of osteocalcin and collagen type I were detected. Each value represents as mean ± S.D. (n = 3), Significant difference from normal control at p^a^ < 0.05, p^c^ < 0.005, p^d^ < 0.001.

MC3T3-E1 cells were treated with various concentrations (1, 10, 100 μg/mL) of morroniside and loganin for 48 h, then the amounts of caspase-3, caspase-9, bcl-2 and RANKL mRNA were assessed by RT-PCR. Each value represents as mean ± S.D.(n = 3), Significant difference from normal control at p^a^ < 0.05, p^c^ < 0.005, p^d^ < 0.001.
